# DFB Lasers Between 760 nm and 16 μm for Sensing Applications

**DOI:** 10.3390/s100402492

**Published:** 2010-03-24

**Authors:** Wolfgang Zeller, Lars Naehle, Peter Fuchs, Florian Gerschuetz, Lars Hildebrandt, Johannes Koeth

**Affiliations:** nanoplus Nanosystems and Technologies GmbH, Oberer Kirschberg 4, 97218 Gerbrunn, Germany; E-Mails: lars.naehle@nanoplus.com (L.N.); peter.fuchs@nanoplus.com (P.F.); florian.gerschuetz@nanoplus.com (F.G.); lars.hildebrandt@nanoplus.com (L.H.); johannes.koeth@nanoplus.com (J.K.)

**Keywords:** DFB, laser, QCL, sensing, PACS 42.55.Px, 42.62.Fi, 42.62.-b

## Abstract

Recent years have shown the importance of tunable semiconductor lasers in optical sensing. We describe the status quo concerning DFB laser diodes between 760 nm and 3,000 nm as well as new developments aiming for up to 80 nm tuning range in this spectral region. Furthermore we report on QCL between 3 μm and 16 μm and present new developments. An overview of the most interesting applications using such devices is given at the end of this paper.

## 1. Introduction

In recent years the importance of lasers in optical sensing has been continuously increasing [1]. Many apparatus use long-known techniques like fluorescence measurements after excitation by photons, e.g., to detect petroleum contamination in soils [2]. Other systems use cavity ring down or cavity leak-out spectroscopy, e.g., for medical applications with CO [3], or use the excitation of rotational-vibrational spectra for in-situ gas analysis [4] or detection of chemical compounds even in the liquid phase [5]. Excellent scientific books on laser spectroscopy [6] and laser in environmental and life science [7] show the growing interest in the scientific and industrial world. International conferences on laser spectroscopy [8], laser field-applications [9] and tunable diode laser spectroscopy (TDLS) [10] have been generating a steady output of excellent publications as well as scientific exchange for years.

Wherever rapid measurement, cost effective monitoring or miniaturization of devices is crucial, the outstanding characteristics of tunable semiconductor lasers show their advantages. In modern environmental analysis these aspects play an important role, hence tunable semiconductor lasers are the workhorse for such applications. Since simple Fabry-Pérot (FP) laserdiodes (LD) show poor tuning behavior and no monomode spectrum, they are not suited for high resolution applications. Such FP LD in an external cavity [11,12] yield good tuning ranges as well as monomode spectra. Such systems are available for high resolution spectroscopy at all wavelengths between 400 nm [13] and 11 μm [14], however, they incorporate mechanical parts which make them hardly usable for industrial environments, for field measurements or in production sites. This problem is overcome by distributed feedback (DFB) lasers.

The key element for monomode emission and the tuning characteristics is the DFB-grating. Through a special technology, based on lateral metal Bragg gratings incorporated in the laser structure, it is nowadays possible to fabricate monomode emitting semiconductor lasers on any III/V-based material, leading to a broad accessible wavelength range from 760 nm up to 3 μm. In this part of the spectrum many industrial and environmental gas species exhibit strong absorption features [15].

**Figure 1.**
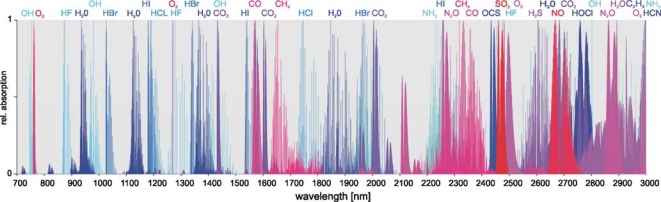
Absorption features of different gases in the 760–3,000 nm wavelength range.

Figure 1 shows absorption features of some exemplary gases in this wavelength range, which is covered by tunable diode lasers made from base-materials like Gallium-Arsenide (760 nm to 1 μm), Indium-Phosphide (1 μm to 1.8 μm) and Antimonides (1.6 μm to 3.0 μm).

Such devices have been used for many years in products for industrial applications, e.g., in such diverse and complicated cases as the detection of alkali atoms in combustion plants [16] or photoacoustic gas sensing with a quartz tuning fork [17], for sensitive and spatially resolved H_2_O vapor detection [18] and for temperature analysis in shock waves [19]. At wavelengths between 3 μm and 16 μm however, even stronger absorption features occur. In this region lead salt LD were used in the past, but they have been largely replaced by quantum cascade lasers (QCL) during the last few years.

As there have been many good review papers about spectroscopy in the past [20–23], we give a brief review about highly reliable semiconductor lasers with DFB structures enabling tunable monomode emission and high optical power in the wavelength range from 760 nm to 16 μm. Typical applications are shown exemplary in the last section.

## 2. DFB Lasers in the 760 nm to 3,000 nm Wavelength Range

There are various ways to realize monomode semiconductor devices, e.g., distributed Bragg reflectors (DBR) [23], external cavity devices [11,12] or DFB LD. The latter exhibit either pure index coupling or—as presented in this paper—complex coupling. Since complex coupled gratings show many advantages over index coupled gratings, a special manufacturing process was developed [25,26] to realize high performance DFB lasers with low threshold currents, high output efficiency and excellent side mode suppression ratios (SMSR).

**Figure 2.**
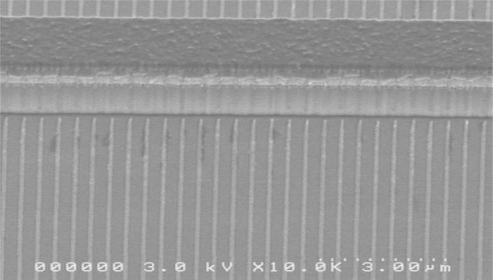
SEM micrograph of a ridge waveguide structure with lateral Cr grating.

The lasers presented in this paper are realized as ridge waveguide structures with a width of 2–10 μm. The feedback grating consists of Cr and is defined laterally to the ridge (see figure 2).

This metallic grating structure results in complex coupling without the need for any overgrowth steps. Since the standard fabrication for DFB lasers requires at least two growth steps, the presented process simplifies the realization of the lasers and reduces defect sources. The biggest advantage of complex coupled lasers over conventional index coupled devices however is the absence of a stopband in the optical spectrum, thus allowing to directly select the Bragg-mode. Furthermore, complex coupled devices benefit from higher mode stability [27–29], lower sensitivity of optical feedback reflections [30,31] and improved modulation properties [32,33]. Since only the evanescent part of the mode interacts with the grating, the spatial overlap is around 10^−4^, two orders of magnitude lower than for an overgrown grating incorporated into the waveguide.

**Figure 3.**
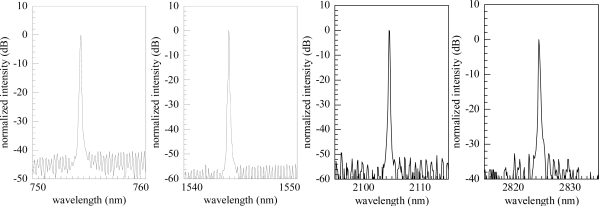
Examples of monomode spectra of laterally coupled DFB lasers with Cr gratings emitting at different wavelengths.

This is compensated by the large imaginary part of the refractive index of the grating metal. The additional losses introduced by the Cr grating lead to only a slight penalty in threshold current and output efficiency [34]. The nodes of the DFB mode are in phase with the absorption grating, resulting in only very small damping of the DFB mode. Typical corresponding optical spectra are shown in Figure 3. The presented process can be used for various material systems, yielding monomode devices in the wavelength range from 760 nm to 3.0 μm. Due to their excellent and customizable properties these devices are covering a wide field of applications from the telecommunications sector to gas sensing, medical and space applications (see Section 5).

**Figure 4.**
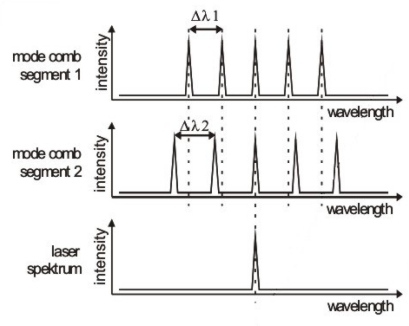
Mode combs of two coupled BSG segments. Lasing wavelength channels can be selected by current tuning of the mode combs and changing the position of maximum overlap.

The presented DFB concept of lateral Cr gratings leading to complex coupling is also a very viable way to realize high quality widely tunable lasers for a large field of different applications. The most convenient way to incorporate the desired multitude of DFB wavelength channels into one device is the concept of binary superimposed gratings (BSG), which were first proposed by Avrutsky *et al.* for use in DBR lasers [35]. For a tunable grating with N optical emission peaks, N binary gratings are virtually superimposed on top of each other, leading to a staircase like grating with N+1 levels which are binarized subsequently [36,37]. Two such gratings yielding comb-like mode spectra with slightly different mode spacing are implemented in two longitudinally coupled laser segments. While the mode spacing in segment 1 is Δ*λ1*, the mode spacing in segment 2 is Δ*λ_2_* > Δ*λ_1_*, thus avoiding the overlap of more than two peaks at one time. By changing the refractive index of a segment, the reflection peaks of the corresponding grating can be shifted. In principle, the refractive index can be adjusted by the temperature and the current. But in general a current-induced tuning of the refractive index is much more convenient and precise than temperature tuning. By shifting the two mode combs of the segments against each other, the Vernier effect can be utilized to select one desired wavelength channel (see Figure 4). If two peaks overlap, the corresponding wavelength is favored by suffering lower internal losses, resulting in monomode emission at this spectral position.

**Figure 5.**
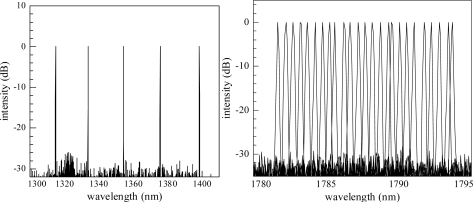
Superimposed emission spectra of BSG LD showing a discrete tuning range of more than 80 nm (left-hand) and a 0.5 nm grid covering 13 nm (right-hand).

A simultaneous tuning of the segment currents results in continuous tuning of the selected wavelength. The continuous tuning range is generally limited to a few nanometers. However, the capability of continuously shifting the wavelength is most important for sensing applications, since it allows for precise scanning of molecule absorption lines. Figure 5 shows two examples of wavelength ranges accessible with such multi-segment DFB BSG lasers. The left-hand picture displays a more than 80 nm wide discrete tuning range in the 1.4 μm region, thus effectively enabling the substitution of five conventional DFB LD with one DFB BSG device. The right-hand picture shows superimposed emission spectra of a BSG LD covering a 13 nm range with 25 different wavelength channels.

**Figure 6.**
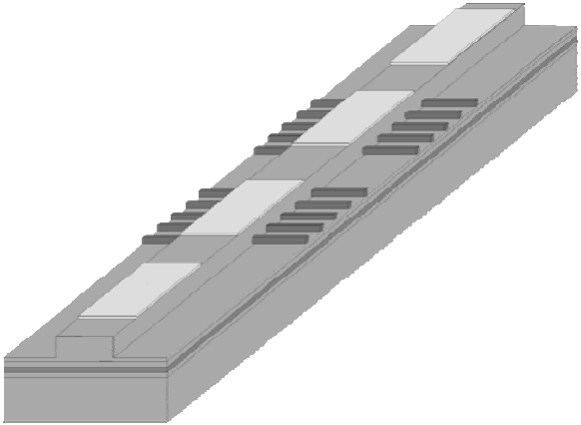
Four segment BSG device. The grating segments allow for wide monomode tunability. The segments without grating allow for current controlled phases.

To improve the selectivity of each mode even further, four segment lasers can be realized (see Figure 6). Two additional current controlled segments without gratings are used to enable phase control and allow for a stable tuning of the wavelength channels by ensuring spectral coinciding of BSG modes and FP modes in the resonator. The overall potential of the concept becomes clear when considering that in general there is no restriction for the number of channels incorporated in one BSG grating, nor for their positions and their spacing. Widely tunable monomode sensing devices covering wavelength ranges of 80 nm (at 1.3 μm) allowing reproducible DFB-like tuning on application-specific wavelength channels with excellent SMSRs are thus attainable. This tuning range exceeds the possibilities of ordinary DFB lasers, which typically show tuning ranges of less than 5 nm, by far.

Thus widely tunable DFB BSG devices are suited excellently for multi component gas analysis since they cover a wide wavelength range which usually contains several absorption lines of the gases of interest. Therefore only one tunable device may replace several conventional DFB lasers, resulting in significantly reduced costs and compact assembly components.

Due to their improved properties in respect to conventional tunable DBR lasers [38–41], these devices represent a convenient as well as highly accurate possibility for multi-component gas analysis. The presented concept of coupled segments involving lateral metal BSG gratings can be applied to virtually every semiconductor material [37], so far enabling devices with emission wavelengths between 760 nm and 3.0 μm.

## 3. Monomode DFB Quantum Cascade lasers in the Mid Infrared Spectral Regime (MIR)

The fundamental rotational-vibrational molecular absorption bands of most relevant smaller molecules are located in the MIR (wavelength range from 3 to 24 μm) [42]. As a result, their line strengths in this region are several orders of magnitude higher than in the NIR, which offers great potential for increased detection sensitivity of trace gases. This makes laser sources in this spectral region very attractive for applications such as in-situ process control, environmental monitoring and biomedical diagnostics for which the development of non-invasive measurements are highly favorable [42,43]. The atmospheric windows at 3–5 μm and 8–12 μm enable other applications like free-space optical communications [44] and infrared countermeasures [45].

Before the first demonstration of QCL in 1994 [46], the only available semiconductor lasers operating in the MIR were lead-salt LD, which suffer from low operation temperatures and therefore need cooling with liquid nitrogen. Over the past 15 years the performance of QCL has greatly improved and operation above room temperature has been demonstrated in the wavelength range from 3.1 to 16 μm [47,48].

The coverage of such a wide spectral region with a single laser type is possible because the stimulated emission in QCL relies on intersubband transitions in heterostructures within the conduction band. Therefore the energy separation of the upper and lower laser level can be tailored to achieve lasing at a designated wavelength [49]. Nowadays QCL operating in pulsed or continuous wave mode are commercially available by a number of suppliers. Mounted on thermo-electrically cooled submounts in standard housings, they are compact, rugged and maintenance-free light sources for research, industrial applications and environmental monitoring in the field or even outer space.

**Figure 7.**
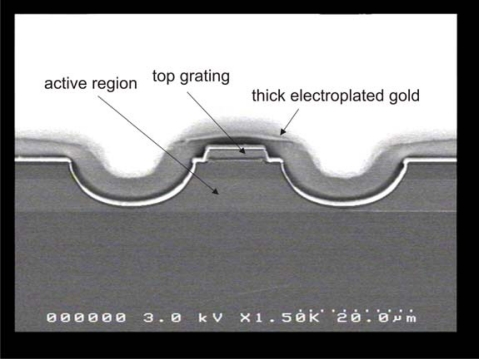
SEM micrograph of the facet of a double channel ridge waveguide with DFB top grating and 5 μm thick electroplated gold.

QCL DFB gratings are most often implemented as either buried gratings [50] or as top gratings [51] usually defined by electron beam lithography and etched into the top of the laser ridge to enforce monomode emission. To provide efficient heat dissipation, laser ridges are generally processed as double channel ridge waveguides with thick electroplated gold around and on top of the laser ridge (see Figure 7) or as buried heterostructure ridges [52]. This is very important for operation at high duty-cycles or in continuous wave mode, because in comparison to diode lasers QCL exhibit considerably higher electrical power consumption and lower wallplug-efficiencies. Thermal management and optimal mounting is therefore indispensible.

The temperature tuning rate of DFB QCL in the MIR depends on the emission wavelength and lies in the range of 0.05 to 0.18 cm^−1^/K. This yields about 4 to 13 cm^−1^ total tuning range on a standard Peltier cooler. Because the spectral gain region can be tailored to be a lot wider (up to 430 cm^−1^ [14]) by incorporating bound to continuum active region designs [53] or multiple heterogeneous cascades [54], a lot of effort has been made to enable monomode emission over the entire available bandwidth. Concepts to extend the monomode tuning range include DFB laser arrays [55], multisegment lasers [56] and external cavity QCL. Especially the latter have shown extreme tuning ranges of up to 3.8 μm [14].

**Figure 8.**
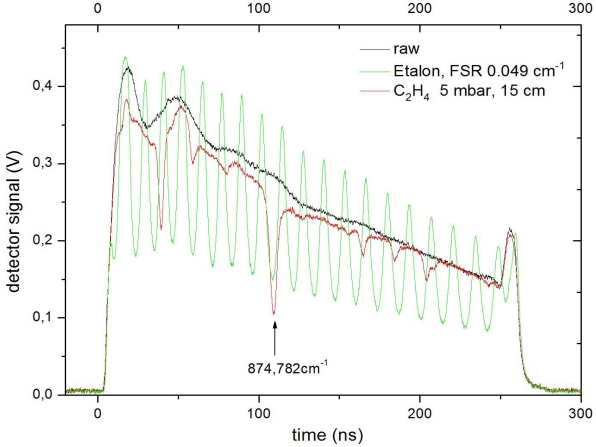
Intra-pulse tuning of a DFB QCL emitting at 11.43 μm driven with 250 ns pulses at room temperature. The black line shows the raw pulse shape. A measurement using an etalon (green line) with a free spectral region (FSR) of 0.049 cm^−1^ indicates a chirp-rate of 0.382 cm^−1^ /100 ns. An absorption experiment (red line) allows for precise determination of the wavelength axis using well known absorption lines of C_2_H_4_.

Users of DFB QCL in gas spectroscopy typically operate pulsed QCL either in intra- or inter-pulse tuning mode to scan across the absorption line of interest. The former method relies on spectral tuning of the DFB mode within a long pulse (usually a few 100 ns) as a result of the current induced temperature rise in the waveguide during a single pulse (see Figure 8). The latter uses short pulses (around 10 ns) and a DC-current ramp below threshold to heat up the laser and provide the desired tuning from pulse to pulse.

**Figure 9.**
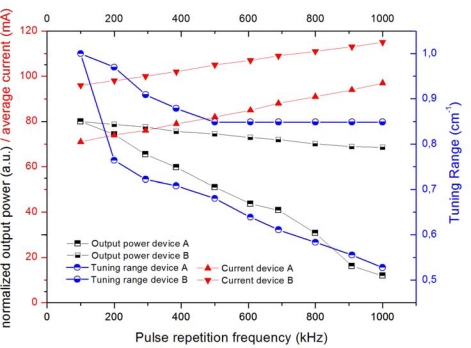
Tuning range, output power and average current at various pulse repetition frequencies (PRF) of two double channel DFB QCL (15 μm ridge width, 2 mm length) at 11.43 μm. They are driven in inter-pulse tuning mode with a pulse length of 8 ns, a cycle frequency of PRF/1000, and ramp duty-cycles of 94.1% (device A) and 99.8% (device B). The heat sink temperature was 10 °C for device A and 23 °C for device B to yield the same spectral position of the DFB laser mode.

Especially inter-pulse tuning introduces a high heat load into the chip, which needs to be dissipated before the next ramp-cycle to get the maximum tuning range. The more efficiently heat can be dissipated, the higher repetition rates for laser pulses and ramp-cycles can be chosen in order to gain higher average output power and therefore greater signal strength. Figure 9 shows the dependency of the available tuning range on the repetition rate in inter-pulse mode for two DFB QCL of matching measurements operating at 11.43 μm. Device A is mounted epitaxial side up on c-mount, device B is mounted epitaxial side down on c-mount with a high reflectivity (HR) coating on its back facet. The positive effect of the epitaxial side down mounting and HR coating can clearly be seen from the stability of the output power and tuning range as the repetition frequency is increased up to 1 MHz.

## 4. Monomode Interband Cascade Lasers in the 3 μm to 4 μm Spectral Range

The wavelength range from 3–4 μm is particularly interesting for several sensing applications, such as the detection of hydrocarbons. As shown in the preceding sections, several well established types of laser sources approach this wavelength range from both ends. However, decreasing hole confinement and increasing Auger recombination in GaSb-based type I interband diodes limit their usability towards higher wavelengths. Intersubband QCL on the other hand approach the mentioned wavelength range from above but suffers from fast phonon scattering losses. Due to these issues, there is a need for a more efficient laser source in the 3–4 μm regime.

The discussed issues can be circumvented by using interband cascade lasers (ICL) [57,58]. These relatively new and technologically demanding laser sources utilize optical transitions between an electron state in the conduction band and a hole state in the valence band in a cascade of Sb-based type-II QW structures. A broken-gap band edge alignment enables the tailoring of the emission wavelength by altering the cascade structures.

Continuous wave operation above room temperature as well as monomode lasing have been observed in ICLs [59], making the concept very feasible for sensing applications between 3 μm and 4 μm.

## 5. Applications

Diode laser based sensing nowadays is a well-established technique with a wide variety of applications in many different branches of industry and science. Because the emission linewidth of complex coupled DFB LD is typically well below 3 MHz, even the fine structures of absorption features—typically exhibiting linewidths of a few GHz—can be resolved, enabling a clear distinction of different gas features or even isotopes during one measurement cycle. Some key applications are presented in the following sections.

### 5.1. Process Control

Process control has evolved as one of the most important fields of application for LD based gas sensing. One interesting sub-category thereof is combustion control. By monitoring the concentrations of gases generated during combustion processes, such as CO, CO_2_, O_2_, NH_3_, NO_x_ and SO_4_, precise information about the conditions inside the combustion chamber can be obtained. This information allows for a very efficient adjustment of the process conditions resulting in less pollutant emission and higher process efficiency. Usually such measurements are performed at some distance to the burning chamber, e.g., inside a chimney or an exhaust pipe. Consequently there is a time offset depending on how long the gases need from the burning chamber to the measurement setup.

Diode laser based gas sensing however can be employed directly in the combustion chamber, hence enabling zero-offset measurements resulting in very fast and efficient process adjustment. Furthermore the gas measurement itself is very fast and selective due to the monomode emission of the DFB laser diodes [60,61]. Using information derived from the temperature dependent broadening of gas absorption lines, even the temperature in the combustion chamber may be monitored in-situ.

Another example that should be mentioned is plasma control. DFB LD and DFB QCL have been shown to be very useful for the in-situ monitoring of industrial etch processes [43,62]. This enables not only the precise control of the gas composition in the reactor ensuring highly reproducible results, but also the detection of the successful removal of layers of the sample as the etching process proceeds and changes of the concentration of volatile etch products occur.

### 5.2. Fire Detection

Since standard fire detectors are point type detectors, they are not applicable inside buildings with very high ceilings or of generally large dimensions. Usually for such environments linear beam detectors are chosen. A light beam is transmitted through the relevant area and a drop in detected power indicates beam obscuration by smoke. Such systems however are also sensitive to fog, dust, small wall movements, *etc.*, therefore often causing false alarms which are not acceptable in many industrial applications.

**Figure 10.**
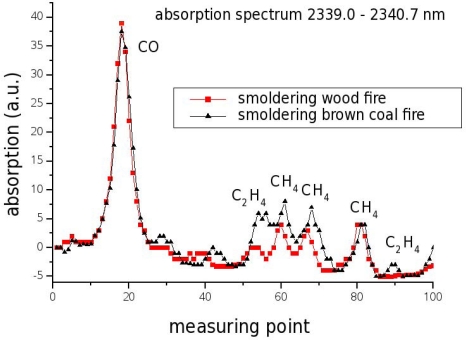
Absorption spectrum for gases generated by smoldering wood respectively brown coal fires [63].

DFB laser based fire detection however bypasses these issues since it is possible to precisely monitor the concentrations of typical fire gases such as CO, CH_4_ and C_2_H_4_ [63,64]. Figure 10 displays the absorption lines of gases generated by smoldering wood respectively brown coal fires. With TDLS it is possible to continuously monitor all these gases in one measurement and even to distinguish between wood and coal fire due to the difference in the C_2_H_4_ signal. The system will only cause an alarm if the ratio between the signal at an absorption peak and the background noise changes, not if the signal strength itself fluctuates. Moreover, such systems are reliable, long-term stable, maintenance-free and can be adapted to individual needs due to the very high selectivity of the measurement.

### 5.3. Space Missions

The compactness and robustness of DFB laser based sensing systems enables their uses even in harsh environments such as outer space. The capacity of TDLS to provide unambiguous information about occurrence and amount of target species and even selected isotope ratios places them in an ideal position to supplement more conventional measurement techniques such as gas chromatography or mass spectrometry [65,66].

### 5.4. Petrochemistry

The petrochemical industry has a high demand for reliable analyzers enabling fast and accurate measurements of various chemical compounds in order to secure the high purity levels necessary to guarantee perfect performance. Concentrations of molecules such as H_2_O, H_2_S, CO_2_ and C_2_H_2_ have to be monitored closely and most notably with as little time offset as possible.

TDLS enables very fast measurement cycles (<1 s) and offers very high measurement resolutions inherent to the system. Furthermore TDLS-based sensor systems require only negligible maintenance and do not need any consumables, so operating costs are very low [61,67]. Because of these advantages, TDLS is ideally suited for petrochemical applications which have been using more conventional analyzing solutions such as gas chromatography, capacitance probes, broadband light spectroscopy or electrolytic moisture analyzers. Those systems however suffer from various drawbacks, e.g., slow update and response time, slow drift in calibration, sensitivity to changing background concentrations and high maintenance and consumable costs.

### 5.5. Medical Applications

Measuring the glucose level is crucial for patients suffering from diabetes. Nowadays the standard measurement procedures require a small blood drop usually drawn by pricking a finger tip each time the glucose level has to be checked, usually up to six times a day. A non-invasive alternative has been developed making use of multiple laser diodes emitting at different wavelengths [68]. The wavelengths are chosen to represent various characteristic positions in the very broad absorption band of glucose. A multiple channels scanning system allows to quantify the glucose level by triggering the different laser diodes sequentially. The light is guided onto the nail bed and conclusions about the glucose level can be drawn from the intensity of the refracted light. Due to the very stable wavelength and power characteristics of complex coupled DFB LD, this approach yields very reliable results providing a practical way to measure the blood glucose levels.

Another major medical application of DFB laser diodes is their use for non-invasive breath tests. The best-known diagnostic test based on this measurement technique is the ^13^C-Urea Breath Test (UBT) used to detect *Helicobacter pylori* infections in humans. For the UBT, the patient orally receives a small dose of urea isotope-marked with ^13^C. If *Helicobacter pylori* bacteria are present in the patient’s stomach, the isotope-marked substance will be split into ^13^CO_2_ and ammonia. The ^13^CO_2_ diffuses via the bloodstream into the patient’s lungs. Consequently it can be detected in the patient’s breath by laser based spectroscopy.

A technique which is ideally suited for this purpose is photoacoustic spectroscopy (PAS) [69,70]. In contrast to transmission spectroscopy, PAS enables offset-free measurements. Furthermore, the relatively long absorption cells usually associated with transmission spectroscopy can be replaced by much smaller ones facilitating the development of small and handy analytical equipment. The breath gas is guided into a gas cell and exposed to the light of a tunable DFB laser source wavelength-matched to an absorption wavelength of CO_2_. The laser light therefore is absorbed by the CO_2_ molecules and transferred into kinetic energy of the surrounding molecules via inelastic collisions. A modulation of the exciting laser source results in local pressure fluctuations *viz.* a sound wave that can be monitored using a microphone and, in some cases, phase sensitive lock in detection schemes. For modulation frequencies below 1 MHz, the photoacoustic signal is directly proportional to the concentration of the absorbing molecules, the absorption cross section of the molecular transition and the intensity of the laser source [70].

DFB lasers offer several advantages over gas lasers often used in PAS measurements. First of all, diode lasers can be modulated directly via the injection current without the use of mechanical choppers, therefore avoiding coherent noise caused by the rotating blade. Furthermore, the frequency shift caused by a current modulation of ±1% is around ±1 GHz, relatively small in comparison with the absorption bandwidth of solids therefore eliminating window signals often deteriorating sensitivity when using a gas laser [71,72].

**Figure 11.**
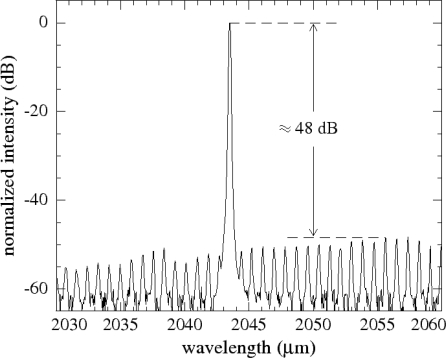
Emission spectrum of a DFB laser diode emitting around 2,043 nm wavelength. The SMSR of the device is >48 dB.

**Figure 12.**
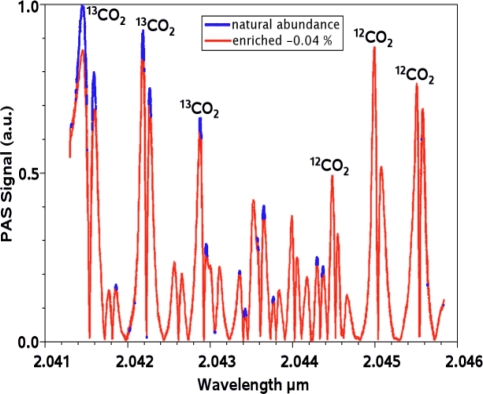
Photoacoustic spectra of CO_2_ in natural abundance (blue line) and with −0.04% enriched ^13^CO_2_ (red line) [70].

As mentioned above, the narrow spectral linewidth of complex coupled DFB lasers enables to resolve different gas isotopes. In the case of UBT measurements, this is taken advantage of in order to discriminate between ^13^CO_2_ and ^12^CO_2_. Figure 11 shows the emission spectrum of a DFB LD emitting right within a rotational-vibrational absorption band of CO_2_ (see Figure 1) at a wavelength of 2,043 nm. The high SMSR of typically >35 dB and the narrow spectral linewidth of <3 MHz guarantee high spectral selectivity and enable cross interference free measurements. Figure 12 shows measurements taken on two samples of CO_2_ with different isotope concentrations using PAS with a current modulation (±5%) induced laser wavelength modulation. The blue line represents CO_2_ in natural abundance (98.42% ^12^CO_2_, 1.10% ^13^CO_2_), the red line a mixture with slightly less ^13^CO_2_ (98.46% ^12^CO_2_, 1.06% ^13^CO_2_). The difference in ^13^CO_2_ concentration results in distinct differences in the measured signal. This test yields a detection limit of about 5 ppm for ^13^CO_2_, thus allowing for detection of variations in ^13^CO_2_ concentration on the order of 1% [70].

Since the level of exhaled nitric oxide is an important indicator for various respiratory diseases, NO is another breath gas often associated with laser based measuring. Due to its strong absorption features in the mid-infrared region above 5 μm, several NO measurement techniques have been developed using DFB QCL devices [73,74]. Such lasers have also been successfully implemented in PAS setups, combining the advantages of PAS detailed above with the long mid-infrared wavelength range accessible to DFB QCL [75,76].

## 6. Conclusions

Tunable DFB lasers are a key feature in a wide variety of different applications ranging from process control to medical diagnostics. Moreover, many of the concepts shown here have left the prototype stage behind and are already implemented in various high impact applications. Nevertheless, there are still a lot of areas where product development has just started, promising many interesting new applications to appear in the next few years. In particular, widely tunable monolithic DFB lasers enabling reduced costs and smaller measurement systems as well as the commercialization of DFB Interband Cascade Lasers are subjects of current development efforts, which will be published in the near future.
